# Protective effects of curcumin against chronic alcohol-induced liver injury in mice through modulating mitochondrial dysfunction and inhibiting endoplasmic reticulum stress

**DOI:** 10.29219/fnr.v63.3567

**Published:** 2019-11-01

**Authors:** Baoying Wang, Xiaolin Gao, Baoguang Liu, Yucheng Li, Ming Bai, Zhenqiang Zhang, Erping Xu, Zhang’e Xiong, Yunlian Hu

**Affiliations:** 1 Key Laboratory for Modern Research on Zhongjing’s Herbal Formulae of Henan Province, Scientific Research and Experiment Center, Henan University of Chinese Medicine, Zhengzhou, China; 2 Basic Medical School, Henan University of Chinese Medicine, Zhengzhou, China; 3 Department of Gastroenterology and Key Laboratory for Molecular Diagnosis of Hubei, The Central Hospital of Wuhan, Tongji Medical College, Huazhong University of Science and Technology, Wuhan, China; 4 Department of Gastroenterology, Hubei Hospital of Traditional Chinese Medicine, Wuhan, China

**Keywords:** curcumin, alcohol, liver injury, mitochondria, endoplasmic reticulum stress, inflammation

## Abstract

**Background:**

Curcumin is a major active ingredient extracted from powdered dry rhizome of Curcuma longa. In Ayurveda and traditional Chinese medicine, it has been used as a hepatoprotective agent for centuries. However, the underlying mechanisms are not clear.

**Objective:**

The present study is to investigate the hepatoprotective effects of curcumin in chronic alcohol-induced liver injury and explore its mechanism.

**Design:**

Alcohol-exposed Balb/c mice were treated with curcumin (75 and 150 mg/kg) once per day for 8 weeks. Tissue from individual was fixed with formaldehyde for pathological examination. The activities of mitochondrial antioxidant enzymes, Na^+^/k^+^-ATPase, Ca^2+^-ATPase, and Ca^2+^Mg^2+^-ATPase, were determined. The level of mitochondrial membrane potential (MMP) and mitochondrial permeability transition pore (MPTP) opening was also determined. The expression of PGC-1α, NRF1, Mn-SOD, GRP78, PERK, IRE1α, nuclear NF-κB, and phosphorylated IκBα was quantified by western blot. The contents of TNF-α, IL-1β, and IL-6 in the liver were measured using the ELISA method.

**Results:**

Curcumin significantly promoted hepatic mitochondrial function by reducing the opening of MPTP, thus increasing the MMP, promoting the activity of Na^+^/k^+^-ATPase, Ca^2+^-ATPase, and Ca^2+^/Mg^2+^-ATPase, and attenuating oxidative stress. Curcumin upregulated the expression of PGC-1α, NRF1, and Mn-SOD, and downregulated the expression of GRP78, PERK, and IRE1α in hepatic tissue. Curcumin also attenuated inflammation by inhibiting the IκBα–NF-κB pathway, which reduced the production of TNF, IL-1β, and IL-6.

**Conclusion:**

Curcumin attenuates alcohol-induced liver injury via improving mitochondrial function and attenuating endoplasmic reticulum stress and inflammation. This study provides strong evidence for the beneficial effects of curcumin in the treatment of chronic alcohol-induced liver injury.

## Popular scientific summary

- In this study, we evaluated the hepatoprotective effects of curcumin in chronic alcohol-induced liver injury and explored its mechanism.
- We found that curcumin protected the liver from damage caused by alcohol via modulating mitochondrial dysfunction.
- Furthermore, curcumin inhibited endoplasmic reticulum stress, and curcumin attenuated inflammation by inhibiting the IκBα–NF-κB pathway.
- Our work provides strong evidence for the benefiial effects of curcumin in the treatment of chronic alcohol-induced liver injury.

Alcoholic liver disease (ALD) is caused by prolonged high alcohol intake. ALD is a complex process that includes a wide spectrum of hepatic lesions, from steatosis to hepatitis, fibrosis, and cirrhosis. Statistics show that in the United States 50% of chronic liver disease deaths each year are caused by excessive drinking; in Europe, ALD is the most common cause of chronic liver disease, accounting for more than 75% of cases of cirrhosis (1). In China, with the acceleration of work stress and changes of lifestyle, alcohol consumption is growing rapidly. ALD becomes the second cause of liver disease, second only to viral hepatitis such as hepatitis A and hepatitis B (2). Therefore, intensive study of the pathogenesis of ALD and treatment has become a hot topic in ALD research.

Recent studies have shown that oxidative stress and endoplasmic reticulum stress (ERS) are the key issues in alcohol-induced liver injury. Ethanol metabolism leads to the accumulation of reactive oxygen species (ROS) (3). Mitochondrial compartments are presumed to be the main source and susceptible target of intracellular ROS. Mitochondrial dysfunction is considered as one of the earliest manifestations of alcohol-induced liver injury (4). Ethanol lowers the mitochondrial membrane potentials (MMP) and induces the opening of mitochondrial permeability transition pores (MPTP) (5), resulting in reduced cellular adenosine triphosphate (ATP) synthesis and increased necrosis (6). Extensive studies have confirmed that ERS participates in the development of ALD (7, 8), while ERS can initiate inflammation (9). The molecular link between ERS responses and inflammatory responses might be mediated by the inositol-requiring transmembrane kinase and endonuclease 1α (IRE1α) (10). Activation of IRE1α by ERS activates NF-κB, which, in turn, activates hepatic macrophages to release inflammatory factors and promotes the development of inflammatory responses in the body. ALD results in increased expression of NF-κB, producing multiple cytokines and participating in the inflammatory response (11).

Curcumin, a biologically active component of turmeric (Curcuma longa), is used as an antioxidant and anti-inflammation treatment in Chinese herbal medicine (12, 13). Previous studies have shown that curcumin and its analogs possess powerful hepatoprotective effects on several hepatotoxins (14), including ethanol (15, 16). However, the existing studies of the underlying mechanism mainly discuss the antioxidative stress effects of curcumin against ALD (17–19). Limited reports on the prevention of ALD by curcumin from the perspective of improving on mitochondrial damage and reducing IRE1α-, IκBα-, and NF-κB-induced inflammation were found. Tiwari demonstrated the involvement of cytokine release and inflammatory signaling in chronic alcohol-induced cognitive dysfunction and also suggested the effectiveness of curcumin in preventing cognitive deficits associated with chronic alcohol consumption (20). It was found that curcumin improved liver histopathology in early stage of ethanol-induced liver injury by reduction of oxidative stress and inhibition of NF-κB activation (21).

Thus, the present study was designed with an aim to investigate the effect of curcumin on chronic alcohol-induced liver injury, mitochondrial damage, and IRE1α-, IκBα-, and NF-κB-mediated inflammatory signaling in mice.

## Materials and methods

### Reagents and materials

Curcumin (purity > 98.0%) was obtained from National Institutes for Food and Drug Control (Beijing, China). Alcohol (Red Star wine, 56% *v*/*v*) was obtained from Beijing Red Star Co. Ltd. (Beijing, China). Detection kits for mitochondria SOD, GSH-Px, MDA, Na^+^/k^+^-ATPase, Ca^2+^-ATPase, and Ca^2+^/Mg^2+^-ATPase were purchased from the Jiancheng Institute of Biotechnology (Nanjing, China). Rhodamine 123 (Rh123) was purchased from Molecular Probes (Hayward, CA, USA). Protein lysate was purchased from Biyuntian Biotechnology Research (Shanghai, China). Total Protein Extraction Kit (P1250) and Nuclear Extraction Kit (P1200) were purchased from Applygen Technologies Inc. (Beijing, China). Primary anti-bodies of PGC-1α, NRF1, Mn-SOD, GRP78, PERK, p-PERK, IRE1α, p-IRE1α, IκBα, p-IκBα, NF-κB p65, and β-actin were from Cell Signaling Technology (Beverly, MA, USA). TNF-α, IL-1β, and IL-6 ELISA kits were purchased from Xinbosheng Co. Ltd (Shenzhen, China). All other chemicals were of analytical grade and were obtained from standard commercial suppliers.

### Animals and treatments

Male Balb/C mice (25 ± 2 g, 6 weeks old) were obtained from the Experimental Center of Medical Scientific Academy of Hubei (Wuhan, China). Mice were housed at 22 ± 2°C, the humidity of 50 ± 5%, and a 12-h light–dark cycle, with free access to food and water. The experiment was approved by the Animal Ethics Committee of Henan University of Traditional Chinese Medicine and met the requirements of the Laboratory Animal Act of the People’s Republic of China. After 1 week of acclimatization, mice were divided randomly into five groups (*n* = 10) – (1) normal control group (olive oil, 10 mL/kg, orally); (2) curcumin control group (150 mg/kg, orally); (3) alcohol model group; (4) alcohol + curcumin (75 mg/kg, orally) group; and (5) alcohol + curcumin (150 mg/kg, orally) group. Except the normal control group and curcumin control group, the other groups were administered intragastrically with red star alcohol (56% Vol) 10 mL/kg for 2 weeks; then, the alcohol dose was increased to 12 mL/kg for 2 weeks; and the next 4 weeks, 15 mL/kg alcohol was given to each mice in groups C, D, and E. Curcumin was dissolved in olive oil. One hour prior to alcohol intake, dissolved curcumin was given to the mice in B, D, and E groups. Twelve hours after the last administration, animals were killed. Livers were totally excised from the mice. One piece of tissues from the same liver lobe in each animal was fixed with 10% formaldehyde for histopathological examination; another piece of tissues from the same liver lobe in each animal was for extraction of mitochondria; and the left liver was for homogenate and protein extraction.

### Histopathological examination

The mice livers from all groups were fixed in 10% neutral paraformaldehyde immediately, and then dehydrated in gradual ethanol (50–100%), cleared in xylene, and embedded in paraffin. The paraffin-embedded specimens were sectioned at 5μm and then stained with hematoxylin and eosin.

### Isolation of mitochondria from liver tissue

Liver mitochondria were isolated with differential centrifugation. First, add 3 mL of sucrose buffer to the centrifuge tube, carefully add 3 mL of liver homogenate along the tube wall to cover the upper layer, and then perform differential centrifugation as described by Patel et al. (22).

### Hepatic mitochondria antioxidative enzymes and lipid peroxides assay

Superoxide dismutase (SOD), glutathione peroxidase (GSH-Px) activities, and malondialdehyde (MDA) level were measured using commercial assay kits; SOD and GSH-Px activities were expressed as U/mg protein; and MDA level was expressed as nmol/mg protein.

### Mitochondrial membrane potential assay

Rhodamin 123 (Rh123) is a cation-lipophilic fluorescent dye that can penetrate cell membranes and acts as an indicator of mitochondrial transmembrane potential. It depends on the mitochondrial transmembrane potential in normal cells to enter the mitochondrial matrix and bind to the inner lining of the mitochondria, at which point the fluorescence intensity is diminished. When the integrity of the mitochondrial membrane is disrupted, Rhl23, which binds to the inner mitochondrial membrane, re-releases from the mitochondria, resulting in an increase in fluorescence intensity. Therefore, the change in fluorescence intensity of Rh123 by a fluorescence spectrophotometer can indirectly reflect the level of MMP. The fresh liver mitochondria dilution (0.5 mg protein/mL) was incubated with 200 nM fluorescent dye Rh123. The changes in the fluorescence intensity before and after the addition of the mitochondria were used to evaluate the level of the MMP (*l*ex = 488 nm/*l*em = 535 nm).

### Determination of mitochondrial permeability transition pore opening

The opening of MPTP was measured by the mitochondrial swelling method. When the MPTP opens, the outside components enter the mitochondria through the pores. Mitochondria swell, and the absorbance at 520 nm decreases. The mitochondria were diluted with a swelling solution (120 mmol/L KCI, 20 mmol/L MOPS, 10 mmol/L Tris-HCl, 5 mmol/L KH_2_PO_4_, pH 7.4) to a protein concentration of 0.25 g/L at 25°C. An amount of 200 μmol/L CaC1_2_ was put into the dilution, and the change in mitochondrial absorbance at 520 nm was observed continuously. This change reflects the degree of mitochondrial swelling, suggesting the opening of MPTP.

### Determination of mitochondrial Na^+^/K^+^-ATPase, Ca^2+^-ATPase, and Ca^2+^/Mg^2+^-ATPase activities

ATPase is present on the membrane of tissue cells and organelles and is a protein on the biofilm. ATPase can decompose ATP to form ADP and inorganic phosphorus, and the amount of inorganic phosphorus can be determined to determine the level of ATPase activity. The activities of Na^+^/K^+^-ATPase, Ca^2+^-ATPase, and Ca^2+^/Mg^2+^-ATPase were detected using commercially available kits according to the manufacturer’s instructions (Xinbosheng Co.).

### Western blot analysis

Total proteins from liver tissue were extracted using Total Protein Extraction Kit (P1250; Applygen Technologies Inc.). Briefly, liver tissue was homogenized in 100–300 μL SDS lysis buffer with 0.1 mM PMSF. After incubation on ice for 15 min, then centrifugation at 12,000 *g* for 15 min, supernatant was collected for the following analysis. Nuclear proteins were extracted using Nuclear Extraction Kit according to the manufacturer’s instructions (P1200; Applygen Technologies Inc.). Briefly, liver tissue was homogenized in 100–300 μL lysis buffer with 0.1 mM PMSF and allowed to swell on ice for 15–20 min with intermittent mixing. Tubes were vortexed to disrupt cell membranes and then centrifuged at 12,000 *g* at 4°C for 10 min. The pelleted nuclei were washed thrice with lysis buffer and resuspended in nuclear extraction buffer and incubated on ice for 30 min. Nuclear extract was collected by centrifugation at 12,000 *g* for 15 min at 4°C. Protein concentration was analyzed using a BCA protein assay kit. Samples (50 μg protein each) were separated by 10% SDS-polyacrylamide gel electrophoresis, and then transferred to polyvinylidene fluoride membranes by electrophoretic transfer. After being blocked in 5% nonfat milk in TBST for 1 h at room temperature, membranes were incubated overnight at 4°C with the primary anti-bodies directed against PGC-1α (1:1,000), NRF1 (1:500), Mn-SOD (1:500), GRP78 (1:2,000), PERK (1:1,000), IRE1α (1:1,000), IκBα (1:1,000), p-PERK (1:500), p-IRE1α (1:500), p-IκBα (1:500), NF-κB p65 (1:800), β-actin (1:1,000) and TBP (1:2,000), followed by HRP-conjugated anti-rabbit or anti-mouse IgG at room temperature for 1 h. Target protein bands were observed by chemiluminescence and tableted by Kodak film. Protein levels were quantified by scanning densitometry and analyzed by Image J (Materialize NV, Leuven, Belgium). All results were normalized to β-actin or TBP expression in each group as a percent of control.

### Enzyme-linked immunosorbent assay

Liver homogenates were prepared, and the levels of TNF-α, IL-1β, and IL-6 in the liver were measured according to the ELISA kit instructions (Xinbosheng Co.). Absorbance values of each well of 96-well plate were read at 450 nm, and the levels of TNF-α, IL-1β, and IL-6 in the specimen were determined by plotting standard curves.

### Statistical analyses

All statistical analyses were performed using SPSS 15.0 software (IBM Corporation). Data are expressed as the means ± SD. One-way ANOVA with Bonferroni *post hoc* was used for the statistical analysis. Differences with *P* < 0.05 were taken as statistically significant.

## Results

### Curcumin improved the alcohol-induced liver histopathology in mice

**Fig. 1:**
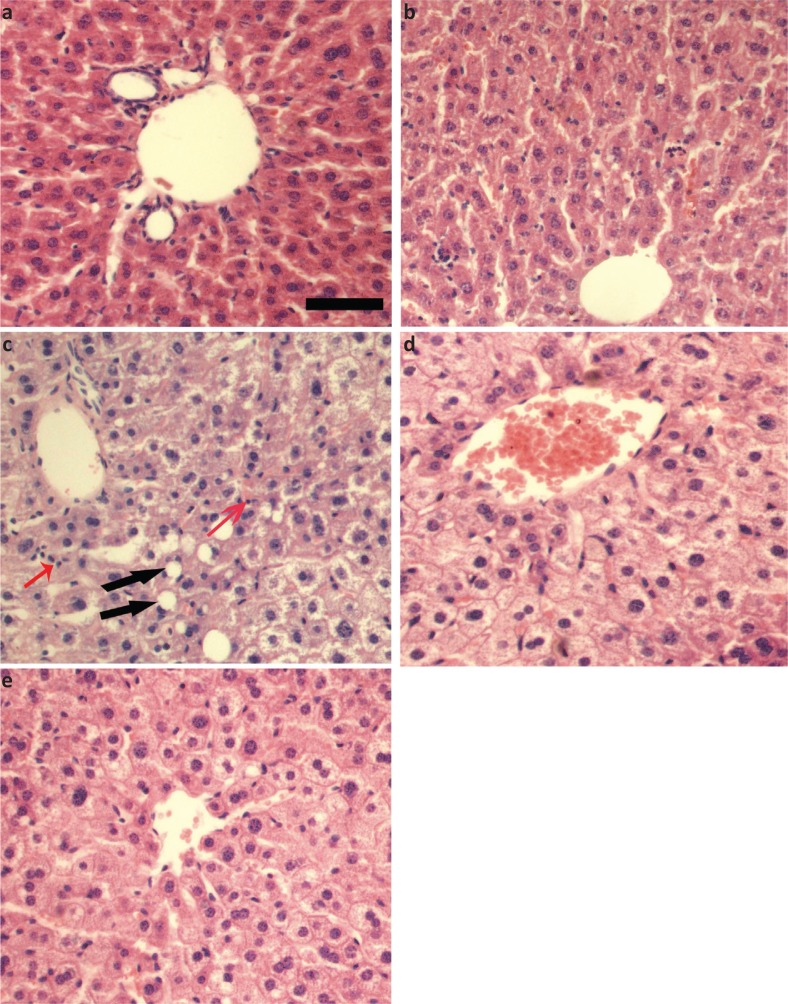
Effects of curcumin on histopathological changes in alcohol-induced liver injury in mice. (a) Normal control group. (b) Curcumin control group. (c) Alcohol model group. (d) Alcohol + 75 mg/kg curcumin. (e) Alcohol + 150 mg/kg curcumin. Liver sections were stained with H&E. Scale bar = 100 μM. Red arrows indicated inflammatory cells and black arrows indicated fat vacuoles.

As shown in Fig. 1, in normal control group and curcumin control group, the structure of the hepatocyte cord was intact, hepatocyte cytoplasm was evenly distributed, and no fat vacuoles and inflammatory cells infiltrations were found (Fig. 1a and b); in the model group, the hepatocytes were swollen, the cytoplasm distributed loosely, and fat vacuoles and inflammatory cells were observed (Fig. 1c); compared with the model group, hepatocyte swelling and cytoplasm looseness were alleviated in curcumin-administered groups, and no fat vacuoles were observed (Fig. 1d and e).

### Curcumin inhibited alcohol-induced liver mitochondria oxidative stress and lipid peroxidation in mice

**Fig. 2:**
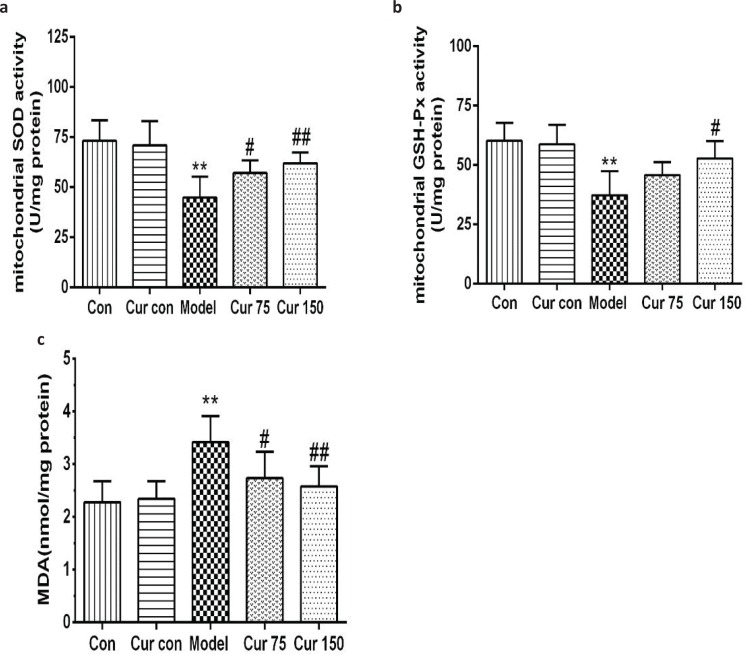
Effects of curcumin on hepatic mitochondrial SOD, GSH-Px activities, and MDA contents in alcohol-induced liver injury in mice. (a) The activity of liver mitochondrial SOD. (b) The activity of liver mitochondrial GSH-Px. (c) The content of liver mitochondrial MDA. ^**^P < 0.01 versus control group; ^#^*P* < 0.05, ^##^*P* < 0.01 versus alcohol model group. Con, normal control group; Cur con, curcumin control group; Cur 75, alcohol+75 mg/kg curcumin; Cur 150, alcohol+150 mg/kg curcumin.

As shown in Fig. 2, compared with the normal control group, the liver mitochondria MDA content was higher (3.42 ± 0.50), and the activities of SOD and GSH-Px were lower (44.83 ± 10.52 and 37.33 ± 10.05, respectively) in the alcohol model group. In contrast, curcumin elevated the activities of SOD (75 mg/kg curcumin: 57.17 ± 6.27, 150 mg/kg curcumin: 62.00 ± 5.40) and GSH-Px (75 mg/kg curcumin: 45.67 ± 5.54, 150 mg/kg curcumin: 52.83 ± 7.33), while reduced the content of MDA (75 mg/kg curcumin: 2.73 ± 0.50, 150 mg/kg curcumin: 2.58 ± 0.38) compared with the alcohol model group (Fig. 2a–c). These data suggest that curcumin might protect the liver against alcohol-induced injury by attenuating oxidative stress and lipid peroxidation.

### Curcumin alleviated the decrease of liver MMP in mice with chronic alcoholic liver injury

**Fig. 3:**
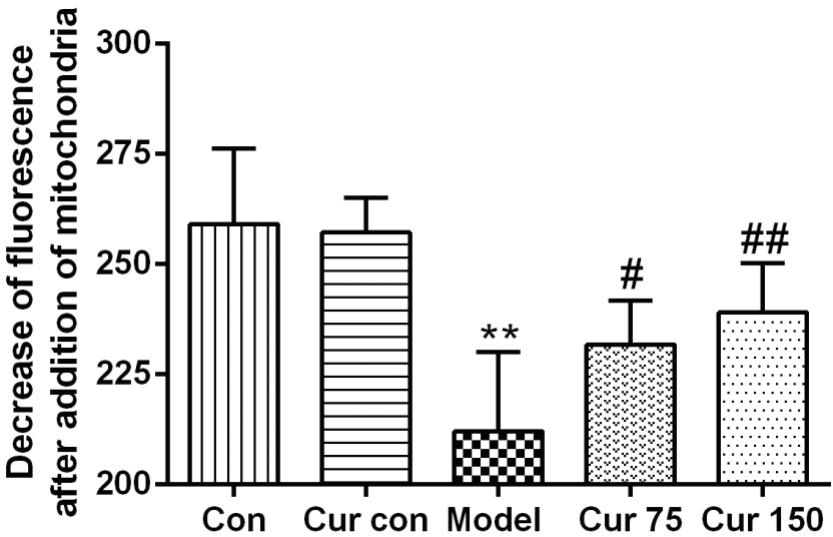
Effects of curcumin on hepatic MMP in alcohol-induced liver injury in mice. ^**^P < 0.01 versus control group; ^#^*P* < 0.05, ^##^*P* < 0.01 versus alcohol model group. Con, normal control group; Cur con, curcumin control group; Cur 75, alcohol+75 mg/kg curcumin; Cur 150, alcohol+150 mg/kg curcumin.

The fresh liver mitochondria were incubated with the fluorescent dye Rh123, and the fluorescence intensity was measured by a fluorescence spectrophotometer. The changes in the fluorescence intensity before and after the addition of the mitochondria were used to evaluate the size of the MMP. As shown in Fig. 3, in the alcohol model group, after the addition of the mitochondria, the decrease of fluorescence remained lower (212.0 ± 17.98) in comparison to the normal control group (259.0 ± 17.16). However, after treatment with curcumin, the decrease of fluorescence increased significantly (75 mg/kg curcumin: 231.7 ± 10.05, 150 mg/kg curcumin: 239.0 ± 11.10) compared with the alcohol model group, which indicates that curcumin leads to the accumulation of Rh123 in the mitochondrial matrix, namely, alcohol decreased the MMP, whereas curcumin increased the MMP.

### Curcumin reduced the opening of MPTP caused by chronic alcohol exposure

**Fig. 4:**
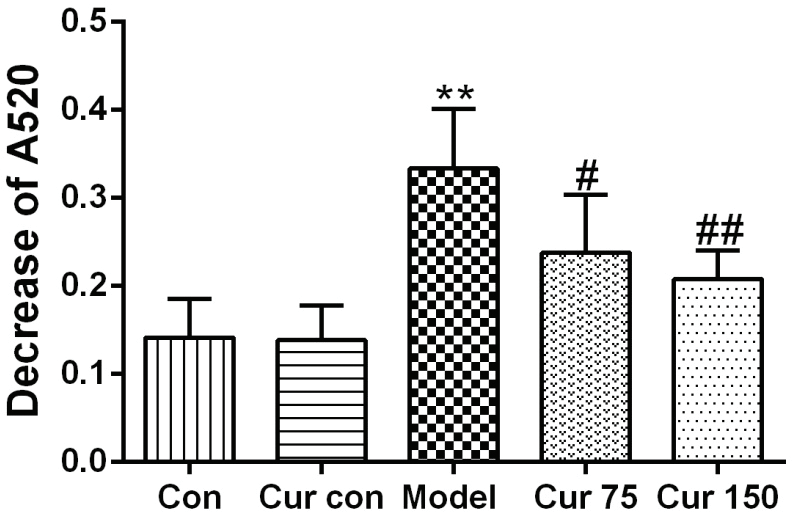
Effects of curcumin on MPTP in alcohol-induced liver injury in mice. ^**^ *P* < 0.01 versus control group; ^#^ *P* < 0.05, ^##^ *P* < 0.01 versus alcohol model group. Con, normal control group; Cur con, curcumin control group; Cur 75, alcohol+75 mg/kg curcumin; Cur 150, alcohol+150 mg/kg curcumin.

The decrease in mitochondrial absorbance at 520 nm reflects an increase in the opening of MPTP. As shown in Fig. 4, after Ca^2+^ was added to the mitochondrial suspension, the absorbance at 520 nm decreased in each group. Compared with the normal control group (0.14 ± 0.04), the decrease in absorbance at 520 nm of the alcohol model group increased significantly (0.33 ± 0.07), which indicates an increase in the opening of MPTP after chronic alcohol exposure. After the intervention of 75 and 150 mg/kg curcumin, the decrease in absorbance at 520 nm was reduced (0.24 ± 0.07 and 0.21 ± 0.03, respectively). Compared with the alcohol model group, the decrease was 28 and 37%, respectively. This result indicates that curcumin can inhibit the MPTP opening caused by chronic alcohol exposure and alleviated the mitochondrial swelling.

### Curcumin alleviated the decreases of the activities of hepatic mitochondrial Na^+^/K^+^-ATPase, Ca^2+^-ATPase, and Ca^2+^/Mg^2+^-ATPase caused by chronic alcoholic exposure

**Fig. 5:**
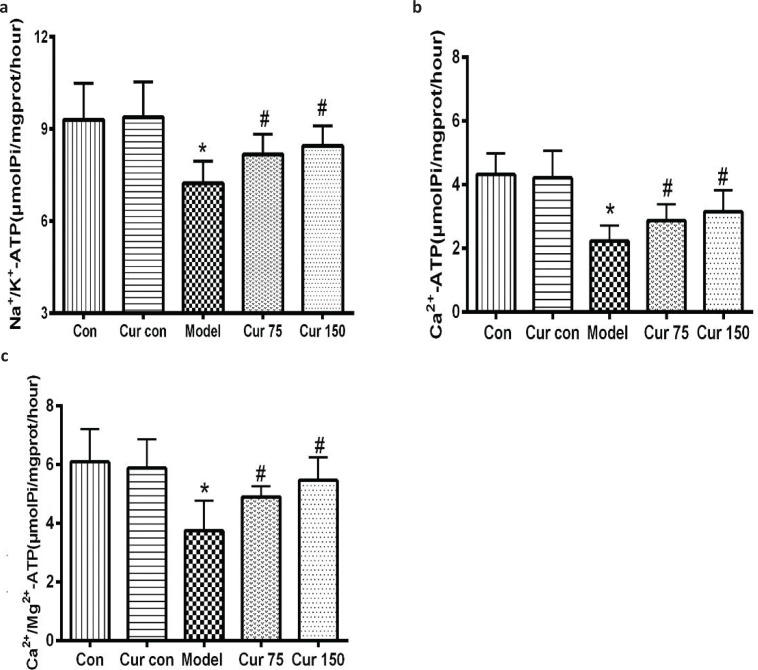
Effects of curcumin on Na^+^/K^+^-ATPase, Ca^2+^-ATPase, and Ca^2+^/Mg^2+^-ATPase activities in the hepatic mitochondria of alcohol-treated mice. (a) The activity of Na^+^/K^+^-ATPase. (b) The activity of Ca^2+^-ATPase. (c) The activity of Ca^2+^/Mg^2+^-ATPase. ^*^ *P* < 0.05 versus control group; ^#^ *P* < 0.05 versus alcohol model group. Con, normal control group; Cur con, curcumin control group; Cur 75, alcohol+75 mg/kg curcumin; Cur 150, alcohol+150 mg/kg curcumin.

As shown in Fig. 5, compared with the normal control group, the activities of Na^+^/k^+^-ATPase, Ca^2+^-ATPase, and Ca^2+^/Mg^2+^-ATPase in the liver mitochondria of the alcohol model group were significantly decreased, and the decline hit 22, 48, and 39%, respectively. After administration of curcumin, the activities of ATPase increased in different degrees. Compared with the alcohol model group, the activity of Na^+^/k^+^-ATPase in 75 and 150 mg/kg curcumin groups increased by 13 and 17%, respectively. The Ca^2+^-ATPase activity increased by 29 and 41%, respectively, and the Ca^2+^/Mg^2+^-ATPase activity increased by 31 and 46%, respectively.

### Curcumin increased the protein expression of PGC-1α, NRF-1, and Mn-SOD in alcohol-induced liver injury in mice

**Fig. 6:**
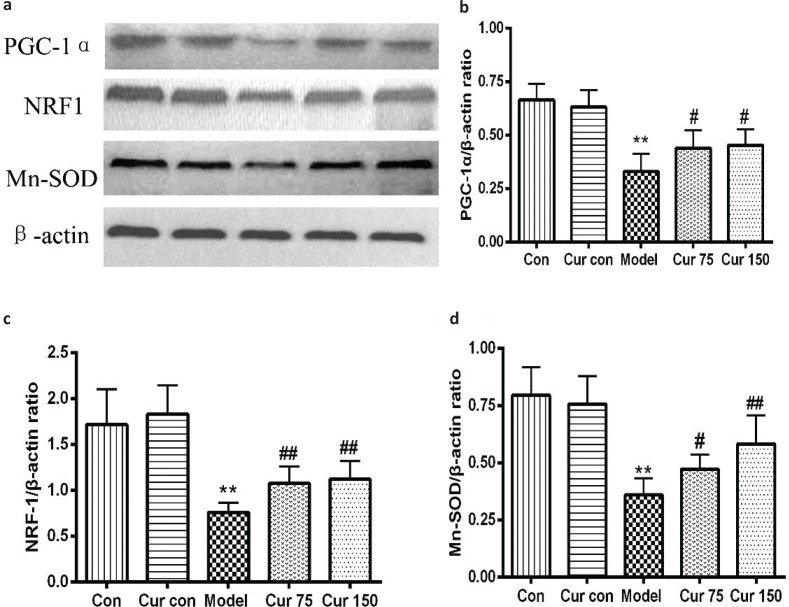
Effects of curcumin on hepatic PGC-1α, NRF1, and Mn-SOD protein expressions in alcohol-induced liver injury in mice. ^**^ *P* < 0.01 versus control group; ^#^ *P* < 0.05, ^##^ *P* < 0.01 versus alcohol model group. Con, normal control group; Cur con, curcumin control group; Cur 75, alcohol+75 mg/kg curcumin; Cur 150, alcohol+150 mg/kg curcumin.

Mitochondrial biogenesis is regulated by PGC-1α and NRF1, so we further investigated the PGC-1α and NRF1 expression response to alcohol exposure. As shown in Fig. 6, compared with the normal control group, exposure to alcohol reduced the expression levels of PGC-1α, and NRF1 proteins in the liver tissues of mice by 50 and 57%, respectively. Mn-SOD is an important antioxidant enzyme which locates in the mitochondria and is capable of reflecting mitochondrial function, especially function of oxidative stress. So, we also measured the expression of Mn-SOD protein in the liver tissues, and it was found that alcohol decreased its expression by 54% in comparison to the normal control group. After administration of curcumin, the expression levels of PGC-1α, NRF1, and Mn-SOD proteins were higher. In comparison to the model group, the expression levels of PGC-1α protein in the liver tissue with curcumin 75 and 150 mg/kg increased by 33 and 37%, respectively; NRF1 protein expression levels increased by 41 and 47%, respectively; and Mn-SOD protein expression levels increased by 31 and 62%, respectively. These results indicate that curcumin can regulate the mitochondrial function and biogenesis.

### Curcumin reduced the protein level of GRP78 and the phosphorylation levels of PERK and IRE1α in mice with chronic alcoholic liver injury

**Fig. 7:**
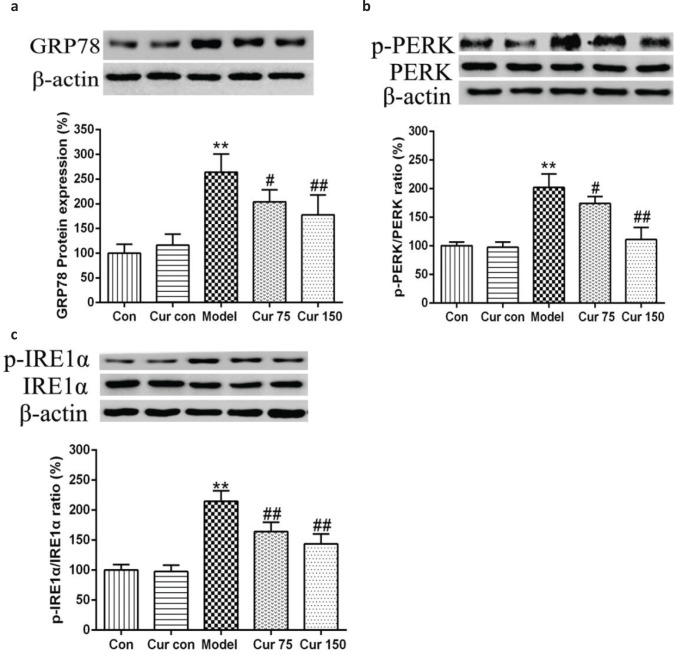
Effects of curcumin on the hepatic GRP78 protein expression and the ratio of p-PERK/PERK andp-IRE1α/IRE1α in alcohol-induced liver injury. ^**^ *P* < 0.01 versus control group; ^#^ *P* < 0.05, ^##^ *P* < 0.01 versus alcohol model group. Con, normal control group; Cur con, curcumin control group; Cur 75, alcohol+75 mg/kg curcumin; Cur 150, alcohol+150 mg/kg curcumin.

To evaluate whether ERS is involved in the development of alcoholic liver injury, we detected the expression level of glucose-regulated protein 78 kD (GRP78), a key signaling molecule in response to ERS. As shown in Fig. 7a, the expression of GRP78 protein was elevated after chronical alcohol exposure (263.90 ± 36.93). Compared with the normal control group (100.00 ± 18.00), it increased by 164%. To confirm whether alcohol also affects the endoplasmic reticulum stressor protein, we measured the phosphorylation of PERK and IRE1α, indicative of their activation. As shown in Fig. 7b and c, compared with control group, chronic alcohol exposure elevated the ratio of p-PERK/PERK andp-IRE1α/IRE1α (202.00 ± 23.87 and 214.60 ± 17.40, respectively). In contrast, curcumin significantly decreased the expression of GRP78 protein (75 mg/kg curcumin: 203.60 ± 24.71, 150 mg/kg curcumin: 177.70 ± 39.90) and the ratio of p-PERK/PERK (75 mg/kg curcumin: 174.00 ± 12.00, 150 mg/kg curcumin: 111.20 ± 21.14) and p-IRE1α/IRE1α (75 mg/kg curcumin: 164.00 ± 15.70, 150 mg/kg curcumin: 143.60 ± 16.50) in the mice’s liver. These results suggest that curcumin can inhibit the activation of ERS and its downstream signaling pathway in alcohol-induced liver injury.

### Curcumin inhibited the activation of NF-kB signaling pathway in alcohol-induced liver injury in mice

**Fig. 8:**
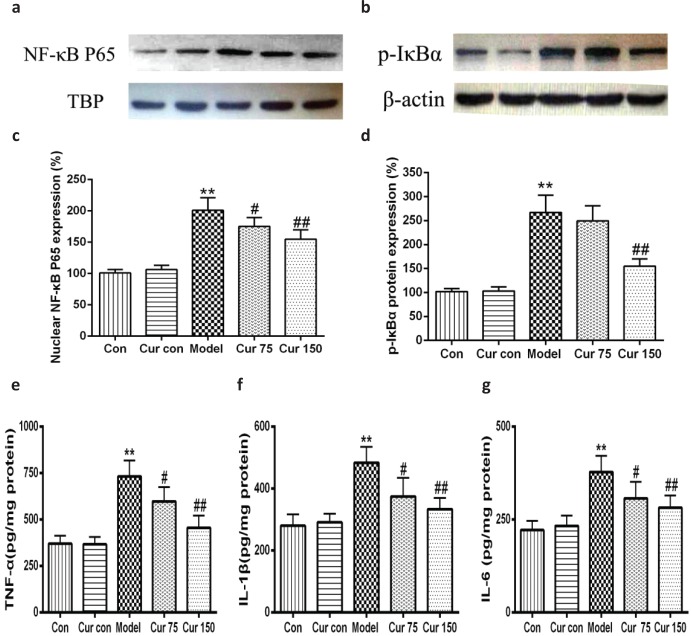
Effects of curcumin on the hepatic IκB α-NF-κB pathway in alcohol-induced liver injury.^**^ *P* < 0.01 versus control group; ^#^ *P* < 0.05, ^##^ *P* < 0.01 versus alcohol model group. Con, normal control group; Cur con, curcumin control group; Cur 75, alcohol+75 mg/kg curcumin; Cur 150, alcohol+150 mg/kg curcumin.

Recent studies have shown that endoplasmic reticulum stress is closely related to inflammatory response. Therefore, we observed the changes of nuclear NF-κB, the phosphorylation of inhibitors of NF-κB (p-IκB). As shown in Fig. 8, following 8 weeks of alcohol intake, livers exhibited marked inductions in the phosphorylation of IκBα (267.00 ± 35.93), along with an increase in NF-κB protein level in nucleus (200.80 ± 19.88), which led to the production of TNF-α, IL-1β, and IL-6 increasing (732 ± 86, 483 ± 52, and 378 ± 44, respectively). After the intervention of curcumin, the levels of nuclear NF-κB and the phosphorylation of IκBα in the liver tissue were decreased significantly (75 mg/kg curcumin: 175.00 ± 14.14 and 249.20 ± 31.52, respectively; 150 mg/kg curcumin: 154.60 ± 15.18 and 153.82 ± 15.22, respectively) in comparison to the alcohol model group, the same with the contents of TNF-α, IL-1β, and IL-6. These results suggest curcumin achieves anti-inflammatory effects by inhibiting the alcohol-induced activation of the IκBα–NF-κB signaling pathway and reducing the production of TNF-α, IL-1β, and IL-6.

## Discussion

Alcohol liver disease, as one of the most common diseases, has become a global threat to human health. Ethanol metabolism leads to the accumulation of ROS and oxidative stress injury. Our previous study showed that alcohol elevates serum ALT and AST levels, and causes lipid disorders (increased TG, TCH, and LDL-C levels and decreased HDL-C level), while curcumin can reverse these alterations in alcohol-induced liver injury in mice by inhibition of oxidative stress via mitogen-activated protein kinase/nuclear factor E2-related factor 2 pathway (23). As mitochondria (through their respiratory chain) and the endoplasmic reticulum (through CYP2E1) are the main sources of ROS (24), the effects of curcumin on mitochondria and endoplasmic reticulum may also be involved in the mechanisms in curcumin treatment of alcoholic liver injury.

Abnormal morphological and functional changes in liver mitochondria have been observed in patients and animal models with ALD. Kurose et al. revealed that after administration of 50 mmol/L alcohol in rats for 30 min, the opening of MPTP in hepatocytes increased and the MMP decreased significantly (25). Yip and Burr confirmed that mitochondrial morphological and functional abnormalities were one of the earliest manifestations of alcohol-induced hepatocyte injury (4). Acute and chronic alcohol exposure changed liver mitochondria structure and function in animal models and humans (26). In the present study, after 8 weeks of alcohol administration, the hepatocyte MMP lowered, the opening of MPTP elevated, and the mitochondrial ATPase activities decreased. In contrast, curcumin exhibited the protection effect of liver mitochondria through the opposite action, which was similar to Bailey’s reports (27). Mitochondrial biogenesis is regulated by PGC-1α and NRF1, which control the transcription and replication of mtDNA and the expression of the mitochondrial respiratory chain complexes (28–30). PGC-1α may stimulate mitochondrial biogenesis and respiration in muscle cells through regulation of NRFs (31). Mitochondrial dysfunctions, such as lipid peroxidation, ATP energy depletion, and ROS overproduction, were significantly improved after high expression of PGC-1α in renal proximal tubule cells (32). Quercetin improved mitochondrial biosynthesis by increasing the expression of PGC-1α and NRF-1 (33). However, alcohol inhibited the expression of PGC-1α in rats (34). Nicotinamide riboside protected against ethanol induced liver injuries via reducing oxidative stress and activating SIRT1-PGC-1α-mitochondrial biosynthesis (35). Our results showed that chronic alcohol exposure not only caused oxidative stress injury (higher liver mitochondria MDA content, and lower activities of SOD and GSH-Px), but also reduced the protein expression of PGC-1α and NRF-1 in the liver. These two aspects may be mutually causal. However, curcumin lowered MDA content, elevated SOD and GSH-Px activities, and increased the protein expression of PGC-1α, NRF-1, and Mn-SOD, which indicated that the mitochondrial antioxidant capacity was not only enhanced from the enzyme activity, but also enhanced from the protein expression level.

Many studies show that ERS participates in the development of ALD. The transcriptional levels of GRP78 and caspase-12 in chronically alcohol-exposed small pigs were significantly elevated, and were positively correlated with hepatic steatosis (36). Mice fed with ethanol intragastrically exhibited an increase in GRP78 and IRE1 (37). Meanwhile ERS is also closely related to the inflammatory response, mainly by inducing nuclear translocation of NF-κB. NF-κB is a key transcriptional regulator that has a central role in the onset of inflammation (38, 39). An increase in the endoplasmic reticulum protein-folding load (e.g., during viral infection) has been shown to result in the activation of NF-κB (40, 41). ERS accentuated myocardial inflammation through the IRE1-associated NF-κB pathway (42). To be more specific, in response to ERS, the autophosphorylation of IRE1α leads to a conformational change, which may bind to the adaptor protein TNF-α-receptor-associated factor 2 (TRAF2) (10); the IRE1α-TRAF2 complex can recruit IκB kinase (IKK), which then phosphorylates IκB and leads to the degradation of IκB and the nuclear translocation of NF-κB (43). Similar results were also reported by Yu (44) and Kitamura (45). Meanwhile IRE1α abrogation blunts the activation of hepatic IKKβ-NF-κB pathway, which leads to reduced production of TNF and IL-6 (46). Li et al. found that curcumin could inhibit p-IRE1α and p-PERK expression in hippocampus CA1 region and attenuate glutamate neurotoxicity by inhibiting ERS-associated inflammasome (47). In our study, we detected the expression level of GRP78 and the phosphorylation levels of PERK and IRE1α in liver tissue by western blot. The expression level of NF-κB in the nucleus of hepatocytes and the phosphorylation level of IκBα were also examined. The results indicated that alcohol exposure increased the expression level of GRP78 protein and the phosphorylation of PERK and IRE1α in mice liver, so did the expression level of nucleus NF-κB and the phosphorylation level of IκBα. In addition, NF-κB promotes the expression of a series of pro-inflammatory cytokines (48). Thereby, we also detected the levels of inflammatory factors, such as TNF-α, IL-1β, and IL-6 in liver tissue, and it was found that in the alcohol exposure groups their levels were significantly increased. However, after treatment with curcumin, the protein expression levels of GRP78 and NF-κB (in hepatocyte nuclei), and the phosphorylation levels of PERK, IRE1α and IκBα all significantly decreased, as were the levels of TNF-α, IL-1β, and IL-6. The above results suggest that curcumin could inhibit the activation of ERS caused by chronic alcohol exposure; it could inhibit the activation of NF-κB inflammatory signaling pathway and reduce the production of inflammatory factors.

## Conclusions

The present study, coupled with our previous report, demonstrates that curcumin attenuates chronic alcohol-induced liver damage probably through multiple targets, among which antioxidative stress, mitochondrial damage repair, inhibition of endoplasmic reticulum stress, and inflammation are involved. Therefore, curcumin as a potential natural anti-ALD drug is worthy of more preclinical and clinical studies.

## Figures and Tables

**Figure:**
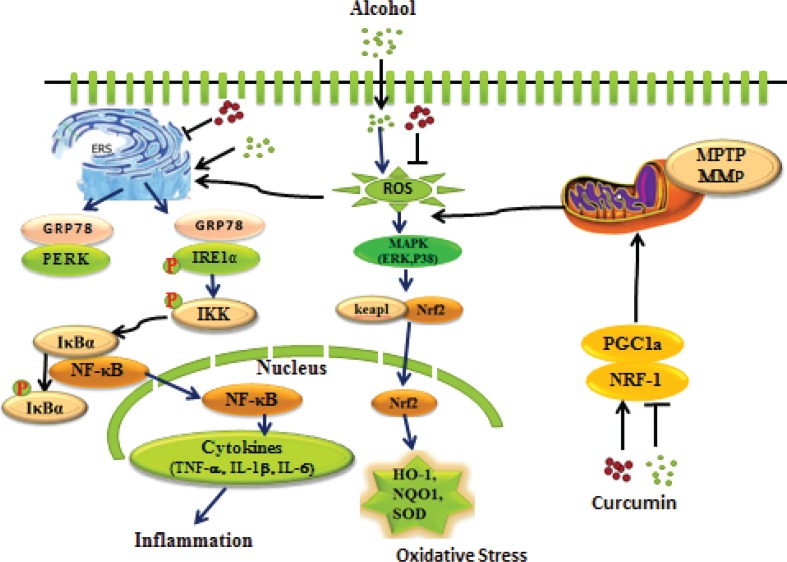


## Graphical Abstract

**The underlying mechanisms involved in the protective effects of curcumin against chronic alcohol-induced liver injury in mice.**

Chronic exposure to alcohol impaired hepatic mitochondrial function, and decreased hepatic protein levels of PGC-1α, NRF1. Meanwhile alcohol-induced phosphorylation of IRE1α and activation of the IκBα–NF-κB pathway, which led to the production of TNF-α, IL-1β, and IL-6.
